# Inference of gene interaction networks using conserved subsequential patterns from multiple time course gene expression datasets

**DOI:** 10.1186/1471-2164-16-S12-S4

**Published:** 2015-12-09

**Authors:** Qian Liu, Renhua Song, Jinyan Li

**Affiliations:** 1 Advanced Analytics Institute, University of Technology Sydney, Broadway, 2007 Sydney, Australia; 2 Advanced Analytics Institute and Centre for Health Technologies, University of Technology Sydney, Broadway, 2007 Sydney, Australia

**Keywords:** gene interaction networks, computational inference, multiple time course gene expression datasets, conserved subsequential patterns

## Abstract

**Motivation:**

Deciphering gene interaction networks (GINs) from time-course gene expression (TCGx) data is highly valuable to understand gene behaviors (e.g., activation, inhibition, time-lagged causality) at the system level. Existing methods usually use a global or local proximity measure to infer GINs from a single dataset. As the noise contained in a single data set is hardly self-resolved, the results are sometimes not reliable. Also, these proximity measurements cannot handle the co-existence of the various *in vivo *positive, negative and time-lagged gene interactions.

**Methods and results:**

We propose to infer reliable GINs from multiple TCGx datasets using a novel *conserved subsequential pattern *of gene expression. A subsequential pattern is a maximal subset of genes sharing positive, negative or time-lagged correlations of one expression template on their own subsets of time points. Based on these patterns, a GIN can be built from each of the datasets. It is assumed that reliable gene interactions would be detected repeatedly. We thus use conserved gene pairs from the individual GINs of the multiple TCGx datasets to construct a reliable GIN for a species. We apply our method on six TCGx datasets related to yeast cell cycle, and validate the reliable GINs using protein interaction networks, biopathways and transcription factor-gene regulations. We also compare the reliable GINs with those GINs reconstructed by a global proximity measure Pearson correlation coefficient method from single datasets. It has been demonstrated that our reliable GINs achieve much better prediction performance especially with much higher precision. The functional enrichment analysis also suggests that gene sets in a reliable GIN are more functionally significant. Our method is especially useful to decipher GINs from multiple TCGx datasets related to less studied organisms where little knowledge is available except gene expression data.

## Background

Gene interactions are indispensable workers in complicated biological processes and molecular functions. This fact highly necessitates a large-scale, system-level view of gene interactions, *i.e.*, gene interaction networks (GINs). A GIN can be represented by a graph with genes as its nodes and gene interactions as its edges. The reconstruction of a *complete *GIN for a species, such as yeast, is too expensive to use wet-lab experiments. As more and more high-throughput genome-wide expression data become available, computational inference of GINs from expression data is critically valuable and feasible with much less cost.

Computational methods for reconstructing GINs from expression data can be categorized into four groups: gene-pair based, single-gene targeted, gene-module based, and integrative methods [1]. **Gene-pair based **methods infer the regulation between a pair of genes [2], usually using Pearson correlation coefficient, Spearman correlation coefficient [3], time-lagged Pearson correlation coefficient [4], or mutual information [5]. **Single-gene targeted **approaches assume that if a target gene is regulated by a group of other genes, the expression levels of the gene could be predicted using the expression levels of that group of genes. Thus, single-gene targeted approaches use the expression levels of a gene as the prediction target and the expression levels of other genes (for example, transcription factors) as features, and learn the relationship of the target genes and other genes using machine learning algorithms and gene-feature selection methods such as different regression methods [3] and Random forest methods [6]. By these two kinds of approaches, gene interactions were determined using all the conditions or time points.

On the other hand, **gene-module based **methods take advantages of biclustering algorithms [7,8] or item-set mining algorithms [9] to detect a cluster of genes which share similar patterns on a subset of gene conditions. Further, **integrative **methods infer gene interactions by combining the knowledge discovered in gene expression data by different methods above [10,11] or with heterogeneous data such as sequence data [8,9]. These methods are able to reconstruct many gene regulations which have been previously detected using wet-lab experiments, for example, in *Escherichia coli*.

However, computational inference of GINs from expression data is still a challenging research problem [3]. For example, in eukaryotes such as yeast, existing methods had very poor performance to decipher gene regulations from gene expression data [3]. It has been reported that the overall performance of gene regulation inference in yeast was quite low [12] and was hardly better than guessing [3,13]. On the other hand, the literature computational methods have their technical limitations [1,3]. For instance, a single dataset is often used which might contain many random gene co-expressions. Another limitation is that many existing methods use global/local proximity measures which are not capable of detecting positive, negative and time-lagged gene correlation at the same time. However in living systems, gene regulations can be positive or negative possibly with time lags, and may also not span all conditions or time points.

To address these issues, we propose to infer gene interactions using a novel subspace pattern--conserved subsequential pattern--from multiple time-course gene expression (TCGx) datasets. Given a dataset, a subsequential pattern contains two subsets of genes such that: (i) the genes within a subset are all positively or time-lag positively correlated with each other on their own subsets of time points, and (ii) every pair of genes--each from a subset--are negatively or time-lag negatively correlated with each other on their own subsets of time points.

We develop an efficient algorithm to detect all subsequential patterns for a TCGx dataset. Based on these patterns, a GIN is built. Assume that a reliable gene interaction would be detected in many times, a reliable GIN is reconstructed using the conserved gene pairs which occur in almost all the GINs of the multiple TCGx datasets. We apply our method on six TCGx datasets related to yeast cell cycle, and validate reliable GINs using the following three sources of experimental knowledge: biopathways from KEGG, protein-protein interaction networks and transcription factor-gene regulations with experimental DNA binding evidences. We also compare reliable GINs with those GINs constructed using a global proximity measure Pearson correlation coefficient on single datasets. This comparison can demonstrate whether the most reliable GINs based on our conserved subsequential patterns achieve much better prediction performance of gene interactions. On the other hand, we investigate the topological properties of the reliable GINs and perform gene functional enrichment test for each gene set whose genes have edges with a same transcription factor in a reliable GIN. All these validations would suggest the usefulness of our algorithm to decipher reliable GINs from multiple TCGx datasets, especially for less studied organisms.

## Methods

### Reconstructing a gene interaction network (GIN) from multiple time-course gene expression (TCGx) datasets

Given *L *TCGx datasets for a same species (*e.g., Saccharomyces cerevisiae*), we use the following steps to reconstruct a reliable GIN. Firstly, we discretize each TCGx dataset to an item sequence database. Secondly, on each discretized TCGx dataset, we define and mine a novel subsequential pattern which is designed to avoid information loss in discretization by using similarity match of items (which is significantly different from identical item match in previous itemset mining algorithms). Thirdly, we develop a new, efficient algorithm to capture all kinds of highly correlated gene pairs, positively correlated, negatively correlated and time-lagged correlated, across subsets of time points. Fourthly, based on these subsequential patterns, we build individual GINs for each TCGx dataset. Finally, we infer a reliable GIN using conserved gene pairs which occur in almost all individual GINs.

### Discretization of a TCGx dataset

Let $M_{N_{V}}\times N_{T}$ denote a TCGx dataset with *N_V _*genes and *N_T _*time points, and *m_i,j _*represents the gene expression level of gene *i *at time point *j*. Let *s_i,j _*be the discretized expression level of gene *i *at time point *j*. In particular,

$$S_{i,j}=\left\{\begin{array}{cc} 3, & \text{if}\;m_{i,j}\geq 2.5*\delta_{i};\\ 2, & \text{if}\;1.5*\delta_{i}\leq m_{i,j}<2.5*\delta_{i};\\ 1, & \text{if}\;0.5*\delta_{i}\leq m_{i,j}<1.5*\delta_{i};\\ 0, & \text{if}\;-0.5*\delta_{i}\leq m_{i,j}<0.5*\delta_{i};\\ -1, & \text{if}\;-1.5*\delta_{i}\leq m_{i,j}\leq -0.5*\delta_{i};\\ -2, & \text{if}\;-2.5*\delta_{i}\leq m_{i,j}\leq -1.5*\delta_{i};\\ -3, & \text{if}\;m_{i,j}\leq -2.5*\delta_{i}; \end{array}\right)$$

*δ_i _*is a value to determine the value range of [-2.5 * *δ_i_*, 2.5 * *δ_i_*] which covers most of expression values of gene *i*. 3, 2, 1, 0, 1, -2, and 3 are seven different discretization items. Then, $S={\left[s_{i,j}\right]}_{N_{V}\times N_{T}}$ is a sequential transaction data set transformed from *M*. In *S*, each gene is represented by a sequence of discretization items of its expressions. The discretization used here is able to eliminate insignificant expression difference in *M*. Note that there is no gold standard to determine the optimal number of discretization items. The more number of discretization items would not capture the similarity between those *m_i,j_*s with insignificant value difference, while the less number of discretization items would lead to poor discrimination between those *m_ij_*s with significant value difference. The number of discretization items should be determined by users based on domain knowledge. Here, we recommend using seven different discretization items according to the understanding of our algorithms developed below.

It is worthwhile to note that some previous works of GIN reference [9] also reconstructed gene interactions after discretization using previous pattern mining algorithms (those items used in previous pattern mining algorithms are called traditional items for an easy reference.). However, those previous pattern mining algorithms failed to pay specific attentions to the difference between traditional items and discretization items from gene expression data. In previous pattern mining algorithms [9], the similarity between different items is meaningless. That is, item '1' is completely different from item '2'. However, discretization items here imply the inherent similarity of original expression levels. For example in a discretized TCGx dataset, 2 is more similar to 3 than 1 is. This similarity is able to avoid some information loss due to the discretization. For example with *δ_i _*= 1, one expression value of *m_i,j _*= 2.49 is discretized to 2, while the other of *m_i,j _m_i',j _*= 2.51 is discretized to 3. If the similarity between item 2 and item 3 is not considered as in previous pattern mining algorithms, this pair of similar expressions is lost during discretization. Meanwhile, 2 is negatively similar to -3, -2 and -1, while 0 is not similar to -3 and -2 either positively or negatively. However, these properties are too difficult, if not impossible, to be considered by previous itemset mining algorithms. In a word, previous pattern mining algorithms would not be able to capture the similarity between these discretization items. A novel pattern taking the similarity above into consideration is necessary.

### Subsequential patterns on a TCGx dataset

*The definition of subsequential patterns: *A novel subsequential pattern is proposed to overcome these limitations in previous itemset mining algorithms. To provide the definition of subsequential patterns, we first define what the similarity between items is.

Let *E *be a set of discretization items {-3, -2, -1, 0, 1, 2, 3}. Two items *e_i _*and *e_j _*in *E *are considered to be similar if and only if *e_j _*- 1 || *e_i _*|| *e_j _*+ 1 or *e_i _*- 1 || *ej *|| *e_i _*+ 1, that is, ||*e_i _*- *e_j_*|| ≤ 1. For instance, 2 is similar to 1, 2 and 3, while -3 is similar to -3 and -2. Based on the similarity, we define a pair-item as *e_i _*:: *e_j _*where *e_j _*= *e_i _*+ 1. Thus, our discretization will result in six pair-items, *i.e*., -3 :: -2, -2 :: -1, ⋯, 2 :: 3. In each pair-item, the two items are assumed to be different. Without loss of generality, the first item in each pair is assumed to be smaller than the second. Please note that -3 :: -1 is not a pair-item. Similarly, two items *e_i _*and *e_j _*are considered to be negatively similar if and only if *-e_j _*- 1 ≤ *e_i _≤ -e_j _*+ 1 or *-e_i _*- 1 *≤ e_j _≤ *-*e_i _*+ 1, that is, ||*e_j _*- *e_i_*||. For example, 3 is negatively similar to -3 and -2, while -2 is negatively similar to 1, 2 and 3.

Further, we say an item *ei *is similar to a pair-item *e_i'_*:: *e_j' _*if and only if *e_i _*is similar to *e_i' _*and *e_i _*is similar to *e_j'_*, e.g., 1 is similar to 1 :: 2 or 0 :: 1. In a similar way, an item *e_i _*is *negatively similar *to *e_i' _*:: *e_j' _*if and only if *e_i _*is negatively similar to *e_i' _*and *e_i _*is negatively similar to *e_j'_*.

Furthermore, *e_i _*:: *e_j _*is similar to *e' *:: *e_j' _*if and only if *e_i _*is similar to *e_i' _*:: *e_j' _*and *e_j _*is similar to *e_i' _*:: *e_j' _*; and *e_i _*:: *e_j _*is *negatively similar *to *e_i' _*:: *e_j' _*if and only if *e_i _*is negatively similar to *e_i' _*:: *e_j' _*and *e_j _*is negatively similar to *e_i' _*:: *e_j'_*.

*A subsequential pattern*is a subsequence of *l_e _*pair-items $\left\{e_{0}::e_{0}+1,\cdots \;,e_{l_{e-1}}::e_{l_{e-1}}+1\right\}$. A subsequential pattern (positively) matches local expressions of gene *i *if there exists 0 ≤ *ii *<*N_T _- l_e _*so that *s_i,ii+jj _*is similar to *e_jj _*:: *e_jj _*+ 1, for all 0 ≤ *jj *≤ *l_e _*- 1. Similarly, a subsequential pattern negatively matches local value movements of gene *i' *if there exists 0 ≤ *ii' *<*N_T _*- *l_e _*so that *s_i',ii'+jj' _*is negatively similar to *e_jj' _*:: *e_jj' _*+ 1, for all 0 ≤ *jj' *≤ *l_e _*- 1. For example, assume a subsequential pattern is {2 :: 3, 1 :: 2, -3 :: -2, 0 :: 1}, *s*_1 _= {0, 3, 2, -2, 0}, *s*_2 _= {0, 0, 2, -2, 0} and *s*_3 _= {-3, -2, 2, 1, 0}, this pattern matches a subset of consecutive values in *s*_1 _with *ii *= 1 and negatively matches a subset of consecutive values in *s*_3 _with *ii' *= 0, but does not match any subset of consecutive values in *s*_2_.

This novel subsequential pattern has several advantages compared with existing works. (i) The subsequential pattern is a local pattern and not required to occur across all time points. (ii) The subsequential pattern is able to detect all positive, negative, time-lagged positive and time-lagged negative correlations of a maximal sublist of genes. Of previous works, some only considered positive correlation between genes, some others found both positive correlation and negative correlation, and some others detected positive correlation and time-lagged positive correlation. None of them could detect a complete set of the subsequential patterns. (iii) Similarity between items retains the inherent similarity of original values which is completely lost in previous works using itemset mining algorithms. (iv) Discretization avoids the effect of insignificant expression difference in bi-clustering algorithms, and at the same time, enables mining a complete set of patterns efficiently rather than heuristic detections in bi-clustering algorithms.

#### The detection of subsequential patterns on a TCGx dataset

We are interested in these frequent subsequential patterns whose length is greater than or equal to a threshold *len*, that is, *l_e _*≥ *len*, and whose support (defined below) is not less than a threshold *sup*. Let a subsequential pattern (positively) match a sublist of genes, called *p*, and negatively match a sublist of genes, called *n*. The support of the pattern is *||p|| *+ *||n||*. It is clear that all genes in *p*(or in *n*) have similar expression levels, while a gene in *p *has oppositely similar expressions to all genes in *n*. Further, a subsequential pattern is closed if there is no more *e_i _*:: *e_j _*added before and/or after the pattern and there is also no more gene inserted in *p *and/or in *n *so that the subsequential pattern still matches all genes in *p *and *n*. Closed subsequential patterns can eliminate redundant subsequential patterns.

Given the discretized version of a TCGx dataset, we develop an efficient mining algorithm to produce all closed subsequential patterns. Our algorithm is based on Apriori [14] with our efficient pruning strategies to speed up the mining process. The naive Apriori algorithm uses deep-first searching: it starts from an empty set, then adds a pair-item *e_i _*:: *e_j _*each time and after that, checks the support of both *p *and *n *for each pattern. In the naive algorithm, many redundant patterns are detected. Thus, we use backward-checking strategy to prune those patterns which have been detected by a pattern occurring before the positions of the patterns, unitize forward-checking strategy to determine whether this pattern is closed, and check items in each position of the pattern to prune duplicate patterns because each item is represented twice by pair-items. Our algorithm is able to efficiently detect a complete and non-redundant set of closed subsequential patterns for all yeast TCGx datasets (described below) in several seconds. The whole framework of the method is given in Algorithm 1. The detail of the pruning strategies is out of the scope of this paper and will not be given here.

In Algorithm 1, given a dataset, pair-items are detected for extending the prefix of a pattern in lines from 3 to 13, while whether a pair-item is frequent is checked in lines from 14 to 16. The closedness of an extended pattern is determined in lines from 17 to 20, and the redundancy of an extended pattern is verified in lines 21 to 24 A pattern would be output if it is closed and non-redundant (in line 25).

**Algorithm 1 **function pApriori(*S_s_*, *prefix-of-pattern*) to detect closed subsequential patterns on a given discretized TCGx dataset

Require:

1) *S_s_*: a given discretized TCGx dataset $S_{N_{V}\times N_{T}}$ with a set of items -3, -2, -1, ⋯, 3

2) *sup*: the minimum number of genes in each pattern

3) *len*: the minimum length of the patterns

4) *max*_0_: the maximum number of insignificant expression levels in each pattern 1: let *I_s _*be a set of six pair-items -3 :: -2, -2 :: -1, ⋯, 2 :: 3.

2: **for all **each pair-item *e_s _*in *I_s _***do**

3:   set both *p *and *n *to {},

4:   **for all **each gene *i *in *S_s _***do**

5:      let *e_i _*be the next item in *i *after *prefix-of-pattern*

6:      **if ***prefix-of-pattern *negatively matches *i *and *e_i _*is negatively similar to *e_s _***then**

7:         put *i *in *n*

8:      **else**

9:         **if ***prefix-of-pattern *(positively) matches *i *and *e_i _*is (positively) similar to *e_s _***then**

10:            put *i *in *p*

11:         **end if**

12:      **end if**

13:   **end for**

14:   **if **||*p*|| + ||*n*|| <**then**

15:      the resultant pattern is infrequent, and thus pruned and continue.

16:   **end if**

17:   determine whether *prefix-of-pattern *∪ *e_s _*is not closed using backward-checking and forward-checking strategies

18:   **if ***prefix-of-pattern *∪ *e_s _*is not closed **then**

19:      call function pApriori(*S_s_*, *prefix-of-pattern *∪ *e_s_*)

20:   **end if**

21:   determine whether *prefix-of-pattern *∪ *e_s _*is duplicate using backward-checking strategy and by checking items in *prefix-of-pattern *∪ *e_s_*

22:   **if ***prefix-of-pattern *∪ *e_s _*is duplicate **then**

23:      the search for *prefix-of-pattern *∪ *e_s _*is pruned, and continue;

24:   **end if**

25:   output *prefix-of-pattern *∪ *e_s _*as a closed non-redundant subsequential pattern

26: **end for**

### Reconstructing GINs using subsequential patterns

Based on these subsequential patterns, individual GINs for a dataset are reconstructed with genes as nodes and gene co-expression in patterns as edges. In an individual GIN, two genes have an edge if they occur in a same pattern of the dataset. Each edge has a label to indicate the interaction is negative, positive or both.

An integrative GIN for multiple datasets is based on all nodes and all edges with the occurrence of edges as weights. Assume that a reliable gene regulation does not occur by chance and appears in almost all datasets, we remove those edges whose weight in the integrative GIN is much less than the number of datasets. The resultant network is called a reliable GIN for further validation. Please note that the weight of positive interactions of a pair of genes is calculated without considering negative interactions of the pair, or vice versa.

As a summary, the whole framework for GIN reconstruction from multiple TCGx datasets is shown in Algorithm 2.

**Algorithm 2 **Inferring a reliable GIN using conserved subsequential patterns from multiple TCGx datasets

Require:

1) *L *TCGx datasets $M_{N_{V}\times N_{T}}$

2) *occ*: the minimum weight of a gene pair in reliable GINs

3) Four parameters for subsequential patterns:

(1) *sup*: the minimum number of genes in each pattern

(2) *len*: the minimum length of the patterns

(3) *max*_0_: the maximum number of insignificant expression levels in each pattern

(4) *i_t_*: the maximum delayed time points allowed

1: **for all **each TCGx dataset $M_{N_{V}\times N_{T}}$**do**

2:   convert *M *into a sequential transaction dataset $S_{N_{V}\times N_{T}}$

3:   use function pApriori($S_{N_{V}\times N_{T}}$, {}) to mine all closed subsequential patterns with at least *len *pair-items and at most *max*_0 _insignificant expression levels (denoted by -1 :: 0 and 1 :: 0) and occurring in not less than *sup *genes

4:   reconstruct a GIN with all genes

5:   add edges for those pairs of genes which occur in a same subsequential pattern and have at most *i_t_*-time-point delay

6: **end for**

7: infer an integrative GIN with all genes

8: add edges for those pairs of genes if they have an edge in a GIN for each of *L *datasets

9: give weights for all edges using their occurrence in GINs for *L *datasets

10: remove those edges whose weights are less than *occ*

11: the resultant network is a reliable GIN

### TCGx datasets

Six TCGx datasets related to yeast cell cycle are used in this work. Their details are presented in Table 1 including the names of the datasets, the number of cell cycles, the time interval to collect gene expression information, and the number of time points. In detail for example, the *elu *dataset [15] involves 14 time points for a cell cycle, and the *cdc15 *dataset [15] involves 24 time points for three cell cycles (*i.e.*, 8 time points per cell cycle); In the *elu *dataset, gene expression information was collected at every 30 minutes, while in the *cdc15 *dataset, gene expression levels were collected at every 10 minutes. Please note that the expression levels of every gene in those datasets were normalized (in those previous works) with the average of the expression levels of a gene close to 0.

**Table 1:** he description of six time-course gene expression (TCGx) datasets.

Dataset	#cell-cycle	time interval	#time-points	reference
elu	1	30 min	14	[15]

alpha	2	7 min	18	[15]

cdc15	3	10 min	24	[15]

cdc28	2	10 min	17	[26]

alpha30	2	5 min	25	[27]

alpha28	2	5 min	25	[27]

'#cell-cycle' is the number of yeast cell cycles in each of six datasets. '#time-points' is the number of time points in each dataset.

Assume that a reliable gene interaction occurs in almost all cell cycles, we consider gene expressions in each cell cycle as an independent dataset (TCGxCC for short). For a TCGx dataset with *h *time points covering two cell cycles, we take the first ⌊*h*/2⌋ + 1 time points as a TCGxCC dataset, and the last ⌊*h*/2⌋ + 1 time points as the other TCGxCC dataset, where ⌊*⌋ is the maximum integer value which is smaller than *. For a TCGx dataset with *h *time points covering three cell cycles, we take the first ⌊*h*/3⌋ + 1 time points as a TCGxCC dataset, the last ⌊*h*/3⌋ + 1 time points as another TCGxCC dataset, and the time points from ⌊*h*/3⌋-*th *to 2*⌊*h*/3⌋-*th *as the other TCGxCC dataset. Thus in the six TCGx datasets, there are in total 12 TCGxCC datasets each of which covers a cell cycle.

These 12 TCGxCC datasets have 3,436 common genes each with less than 2 missing values. The missing value *m_i,j _*of gene *i *at time point *j *is considered to be the average of the most adjacent *m_i,j' _*and *m_i,j" _*where *j' *≺ *j *≺ *j"*, and *m_i,j' _*and *m_i,j" _*are not missing values; if there is no *j' *(i.e., all values of gene *i *before *j" *are missing), *m_i,j _*is set as *m_i,j"_*, while if there is no *j" *(i.e., all values of gene *i *after *j' *are missing), *m_i,j _*is set as *m_i,j'_*.

The parameter settings of our methods are given below for mining interesting subsequential patterns. In the transformation of each of the 12 datasets into a discretized dataset, *δ_i _*is set to the biggest 80%-*th *value of absolute expression levels of gene *i *for discretizing gene *i*. When subsequential patterns are detected on the discretized version of each of the 12 datasets, *sup *is set to 5, and *max*_0 _to 60%, indicating not more than 60% insignificant expression denoted by pair-items -1 :: 0 and 1 :: 0; *len *is set to 25% multiplied by the number of time points in each cell cycle. This is because the number of time points per yeast cell cycle is small ranging from 8 to 14, and a yeast cell cycle usually has several stages, such as G1, S, G2 and M; thus, many gene regulations cannot be expected to occur across a whole yeast cell cycle. To build reliable GINs, *occ *is set to 11, indicating that gene pairs in reliable GINs occur in almost all individual GINs for each yeast cell cycle. At the same time, 1-time-point delay is allowed in subsequential pattern mining for those TCGxCC datasets with more than 12 time points; otherwise, no delayed time point is allowed because of the less number of time points in each yeast cell cycle.

### External validation data

Three kinds of external data are used to validate inferred reliable GINs: protein-protein interaction networks, biopathways and transcriptional regulatory networks.

#### Protein-protein interaction networks

Protein-protein interactions are collected from three sources, DIP [16,17] and STRING [18] (Experimental protein-protein interactions are used with a score cut-off greater than 700 [19]). Compared with TCGxCC datasets, there are 3,101 genes in common with 28,881 protein-protein interactions. A protein-protein interaction is reconstructed if its two proteins have an edge in the reliable network.

#### Biopathway data

The data of biopathways in *S. cerevisiae *is downloaded from the public repository IntPath [20]. This dataset integrates pathway data from several major public databases such as KEGG, WikiPathways, BioCyc and so on. This data and TCGxCC datasets have 3,436 genes in common. We assume that a gene has a strong relationship with another gene in a same biopathway. Then, an edge in reliable GINs is a true positive prediction if the two genes of the edge are in a same biopathway.

#### Regulation networks

Two datasets of transcriptional regulatory networks for yeast are used as external validation standard. One is TNET [21]. This dataset contains 157 transcription factors, and 12,873 regulatory interactions. The other is downloaded from the YEAS-TRACT database [22]. This dataset includes 171 transcription factors, and 41,650 regulatory interactions. These regulatory interactions are determined according to DNA binding evidences which are obtained from wet-lab experiments, such as site-directed mutation of transcription factor binding sites in its promoter region, ChIP, ChIP-on-chip, ChIP-seq and so on.

The two datasets have 11,018 interactions in common. Merging the two datasets together, there are 193 transcription factors and 43,511 regulatory interactions. Of them, genes in 15,356 regulatory interactions and 122 transcription factors are also in TCGxCC datasets. Given a reliable GIN, the edges remain only between those transcription factors and those genes in the regulation data. These regulations with common genes and transcription factors are used in our validation. An edge in reliable GINs is a correct prediction if this edge is also in the regulation data.

### Evaluation measures

Given an external experimental data, assume that *N_cp _*of *N_p _*edges with common genes in an integrative GIN are correct predictions. We use *precision*=*N_cp_/N_p _*to see the percentage of correct interaction prediction in an integrative GIN. Meanwhile, we also use *recall*=*N_cp_/N_e_*, where *N_e _*is the number of gene interactions in an external data, to see the fraction of gene interactions are inferred by an integrative GIN. There are two kinds of *recall*. On one hand, *N_e _*is the total number of gene interactions in an external data, indicating a global *recall*; on the other hand, *N_e _*is the number of interactions in an external data only for those genes are in an integrative GIN, suggesting a local *recall*. Please note that when different predicted GINs are evaluated, *Ne *for local *recall *might change but *N_e _*for global *recall *does not change and keeps the same. Generally, the global *recall *is smaller than the local one. The local *recall *is also meaningful because not all genes are involved in a biological process/molecular function. We use both kinds of *recall *in this work.

## Results and discussion

In this section, we evaluate reliable GINs using the three kinds of external experimental data: protein-protein interaction networks, biopathways and transcriptional regulatory networks. We also investigate the degree distribution of all genes in GINs to see whether it is scale-free. Meanwhile, we perform gene functional enrichment test for regulated genes which have edges with a same transcription factor in a reliable GIN.

### Inference performance by integrative reliable GINs

We vary the different thresholds, *occ*=11 or 12 and the percentage of *len *from 0.25 to 0.3 and then to 0.35, to build integrative reliable GINs with different numbers of edges and of genes. Generally, a smaller threshold results in a larger GIN, while a larger threshold produces a smaller but more reliable GIN. To show the usefulness of our method, we also develop baseline GINs using PCC on the first cell cycle data of the *elu *data (called PCC 1CC elu GIN for short), of *cdc28 *(called PCC 1CC cdc28 GIN for short) and of *alpha30 *(called PCC 1CC alpha30 GIN for short). These data are used for inferring GINs because they are obtained by different biologists. We compare the inference performances by integrative reliable GINs and GINs produced by PCC on the three TCGxCC datasets.

#### Inference performance of protein-protein interactions

**Figure 1:**
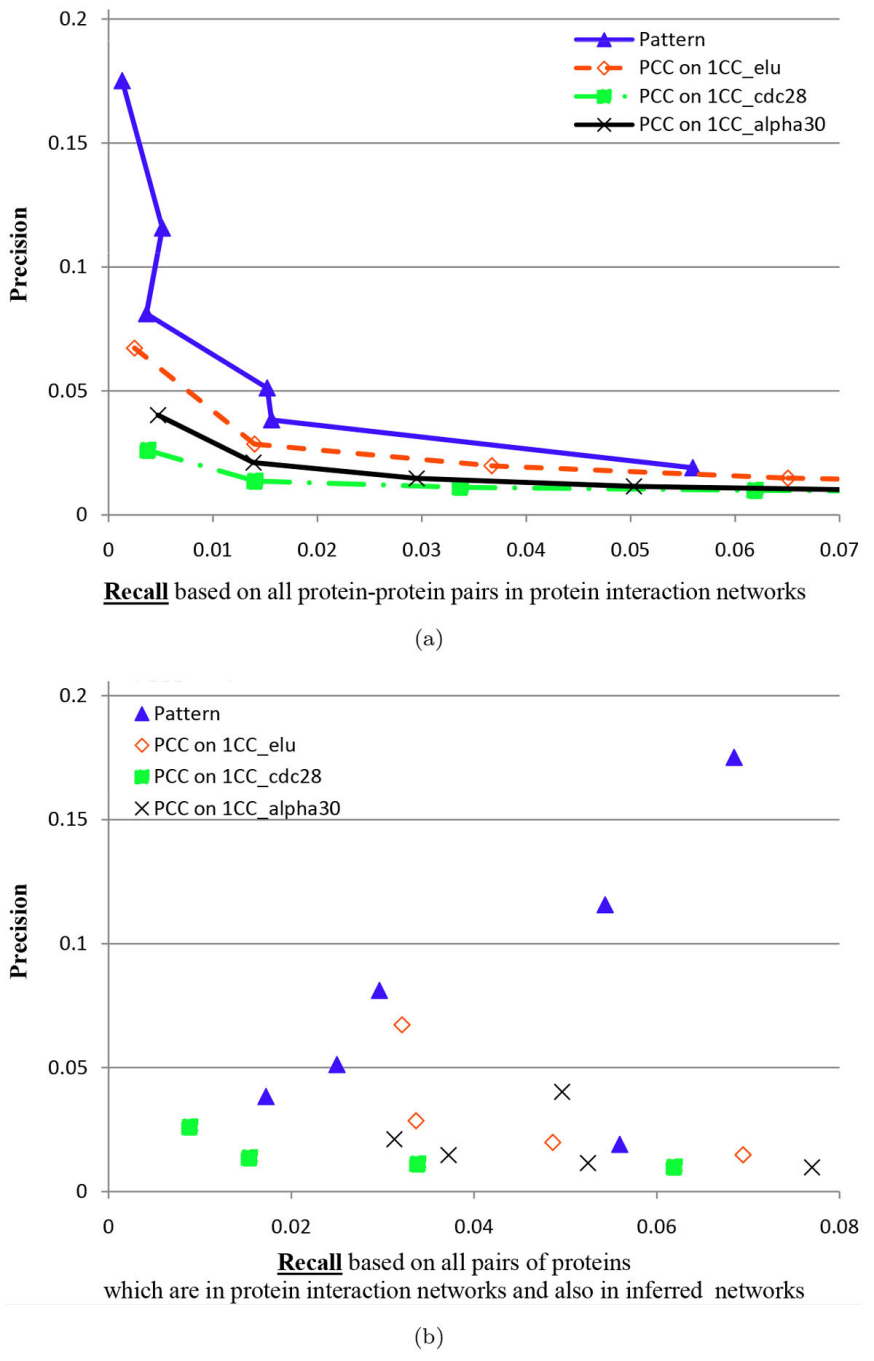
**The evaluation based on protein-protein interactions for two kinds of inferred GINs: one kind using our framework, and the other using PCC on the first TCGxCC datasets (i.e., the first cell cycle data) of elu (1CC elu for short), cdc28 (1CC cdc28 for short) and alpha30 (1CC alpha30 for short)**. 'Pattern' represents an integrative reliable GIN. The subfigure (a) uses global recall, while the subfigure (b) uses local recall. Global recall usually decreases when *occ *increases from a smaller value (11) to a larger value (12) and *len *from 0.25 to 0.3 and then to 0.35, while local recall does not have this relation. Thus, there are lines and curves in (a), but not in (b).

The inference performance of protein-protein interactions is shown in Figure 1(a) and Figure 1(b). It is not surprised that the integrative GINs have higher precision than the PCC 1CC elu GINs. In particular, the most reliable integrative GIN has the precision of 17.5%, which is much higher than the precision (6.7%) of the most reliable PCC 1CC elu GIN at a similar level of global recall. Our integrative GINs also achieve much better performance than the PCC 1CC cdc28 GINs and the PCC 1CC alpha30 GINs. In the most reliable integrative GIN, there are 217 inferred gene interactions where 38 interactions are in the protein-protein interaction networks, while in the second most reliable integrative GIN, there are 1,288 gene interactions where 149 inferred interactions are in the protein-protein interaction networks, resulting in a 11.5% precision.

#### Inference performance of gene co-occurrence in a same biopathway

**Figure 2:**
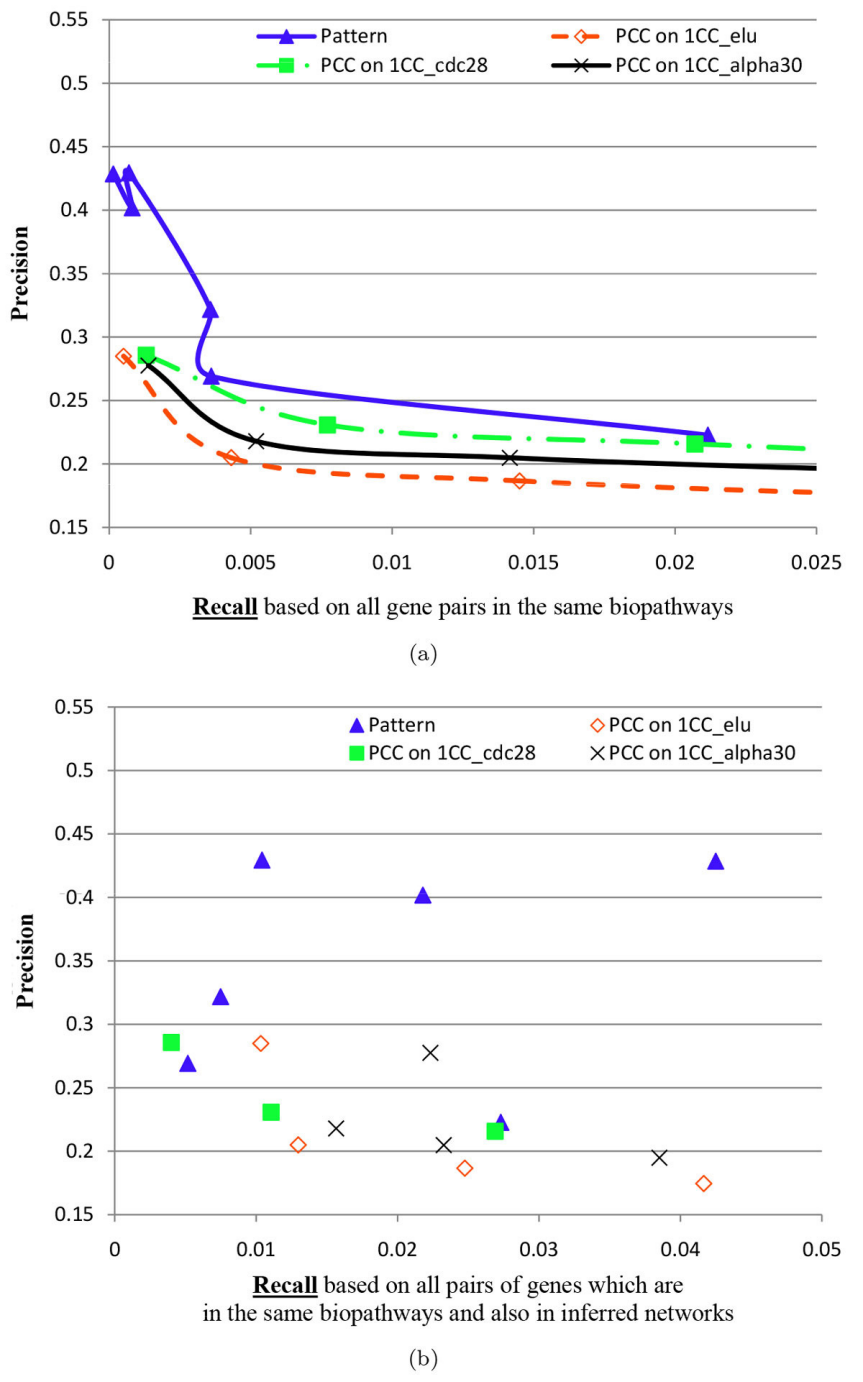
**The evaluation using gene pairs occurring in a same biopathway for two kinds of inferred GINs: one kind using our framework, and the other using PCC on the first TCGxCC datasets (i.e., the first cell cycle data) of elu (1CC elu for short), cdc28 (1CC cdc28 for short) and alpha30 (1CC alpha30 for short)**. 'Pattern' represents an integrative reliable GIN. The subfigure (a) uses global recall, while the subfigure (b) uses local recall. Global recall usually decreases when *occ *increases from a smaller value (11) to a larger value (12) and *len *from 0.25 to 0.3 and then to 0.35, while local recall does not have this relation. Thus, there are lines and curves in (a), but not in (b).

The co-occurrence of two genes states that the two genes are in a same biopathway. We compared gene interactions in referred GINs with gene co-occurrence in biopathways, as shown in Figure 2(a) and Figure 2(b). It is suggested that the integrative GINs always have better inference than the PCC 1CC elu GINs, the PCC 1CC cdc28 GINs and the PCC 1CC alpha30 GINs. In particular, the top three reliable integrative GINs have the precisions higher than 0.4, suggesting a high opportunity of inferred gene interactions to co-occur in a same biopathway.

We also find that although there are 276 biopathways in total, correctly inferred gene interactions in reliable networks mainly occur in 17 biopathways as shown in Table 2. It is interesting to note that several biopathways, such as *'Cell cycle - yeast'*, *'Ribosome biogenesis in Eukaryotes' *and *'Meiosis - yeast' *which are specific to yeast cell cycle, have more correct predictions of gene interactions and were ranked 2nd, 3rd and 5th, respectively, in Table 2 according to the number of the correct predictions.

**Table 2:** The number (indicated in the second column in a descending order) of interactions of genes which co-occur in those biopathways.

Pathway name	# of gene interactions
Metabolic pathways	192

Cell cycle - yeast	69

Ribosome biogenesis in Eukaryotes	42

Ribosome	33

Meiosis - yeast	31

Biosynthesis of secondary metabolites	27

Cell Cycle and Cell Division	24

Purine metabolism	21

Cytoplasmic Ribosomal Proteins	20

DNA replication	20

Pyrimidine metabolism	16

Nucleotide excision repair	10

Mismatch repair	8

Protein processing in endoplasmic reticulum	8

Base excision repair	7

N-Glycan biosynthesis	6

Oxidative phosphorylation	5

The reliable GIN used here is associated with *occ *= 12 and the percentage of *len *equal to 0.25.

#### Inference performance of transcription factor-gene regulations

**Figure 3:**
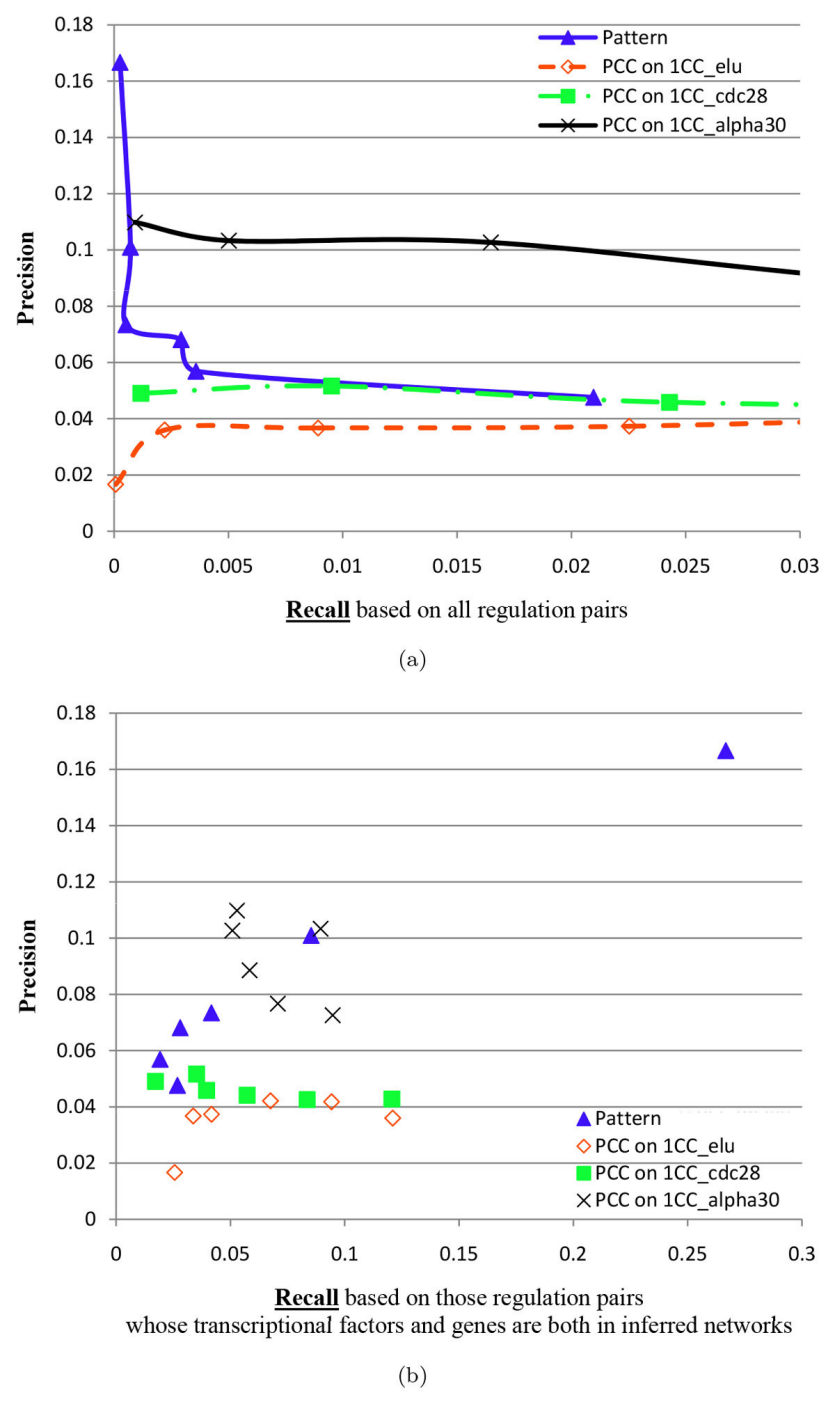
**The evaluation using regulatory interactions between transcription factors and genes for two kinds of inferred GINs: one kind using our framework, and the other using PCC on the first TCGxCC datasets (i.e., the first cell cycle data) of elu (1CC elu for short), cdc28 (1CC cdc28 for short) and alpha30 (1CC alpha30 for short)**. 'Pattern' represents an integrative reliable GIN. The subfigure (a) uses global recall, while the subfigure (b) uses local recall. Global recall usually decreases when *occ *increases from a smaller value (11) to a larger value (12) and *len *from 0.25 to 0.3 and then to 0.35, while local recall does not have this relation. Thus, there are lines and curves in (a), but not in (b).

The inference of transcription factor-gene regulations from gene expression data is an extremely challenging problem in yeast. It is reported in existing works [3,13] that the performance was hardly better than guessing. This conclusion is again confirmed by the PCC 1CC elu GINs, as shown in Figure 3(a) and Figure 3(b), whose precision (less than 0.04) is worse than guessing (0.04). In contrast, integrative reliable GINs have better performance than the PCC 1CC elu GINs and the PCC 1CC cdc28 GINs. In some smaller integrative reliable GINs, the precisions are even higher than 0.1.

However, PCC performs best on the first TCGxCC dataset of *alpha30 *when evaluated using all pairs of transcription factors and genes (in Figure 3(a)), but PCC has much poor performance on the first TCGxCC datasets of *elu *and of *cdc28*. This suggests that the performance of PCC heavily depends on datasets and the dependence is hard to investigate.

On the other hand, when PCC is evaluated using those pairs of transcription factors and genes which are in inferred GINs, integrative reliable GINs achieves comparable performance with the PCC 1CC alpha30 GINs (in Figure 3(b)). Integrating the evaluation on all the three kinds of external experimental data, integrative reliable GINs outperform those GINs produced by PCC.

### The property of reliable GINs

**Figure 4:**
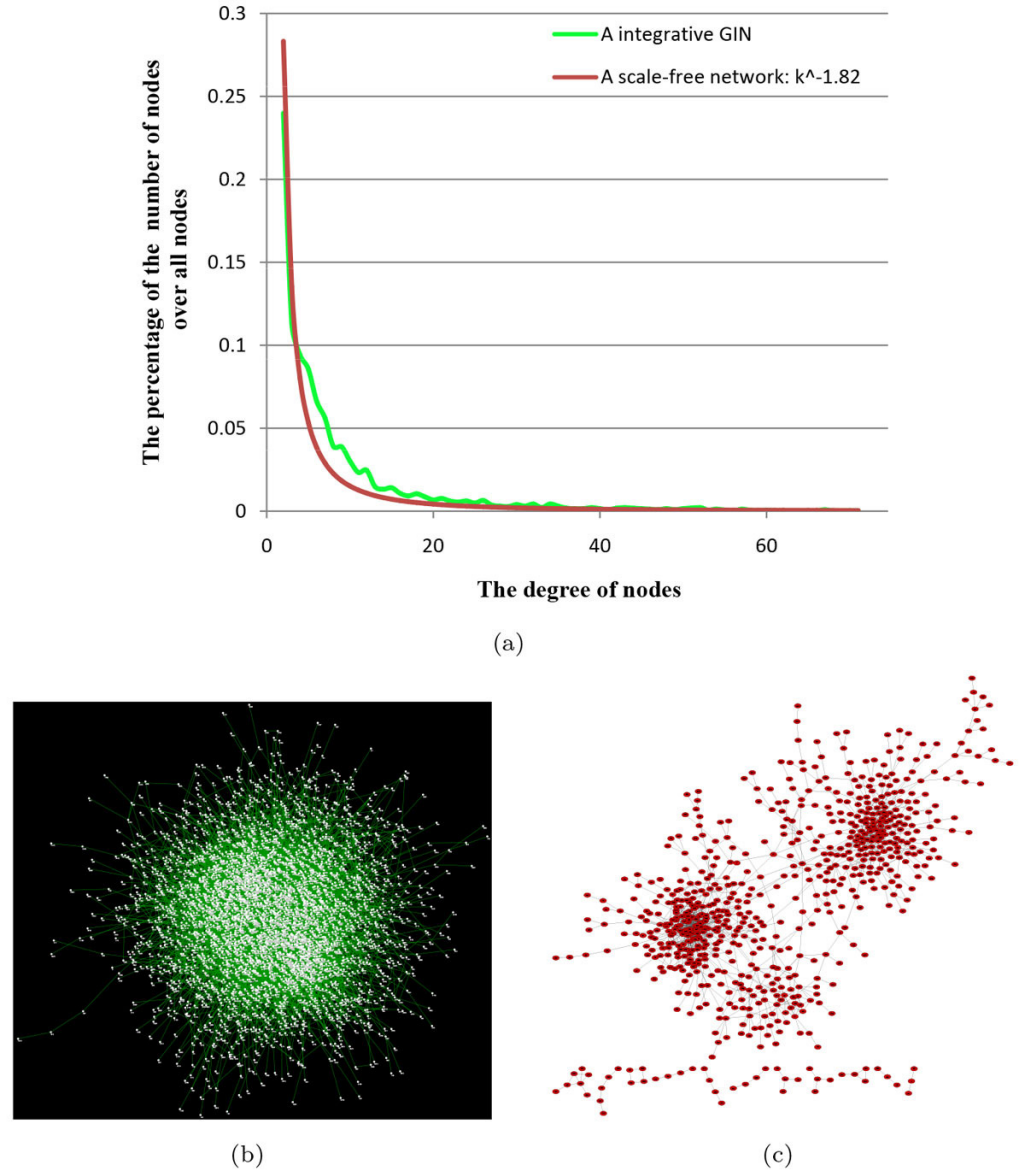
**The distribution of gene interactions in integrative reliable GINs**. (a) The degree distribution of genes in the integrative reliable GIN with *occ *= 12 and the percentage of *len *equal to 0.25. (b) The network view of the integrative reliable GIN with *occ *= 12 and the percentage of *len *equal to 0.25. (c) The network view of the smaller integrative reliable GIN with *occ *= 12 and the percentage of *len *equal to 0.3. This smaller GIN is for better view only. Each node in (b) and (c) represents a gene, and an edge denotes a gene interaction.

In the reliable GIN with *occ *= 12 and the percentage of *len *equal to 0.25, there are 13,996 gene interactions. We then make statistics of the degrees of genes in this GIN. The result is shown in Figure 4(a). It seems that there is a scale-free distribution of the number of nodes which have the same degrees. This distribution is very similar to a scale-free network of *p*(*k*) = *k*^-1.82 ^where *k *is the degree of a node and *p*(*k*) is the percentage of the nodes with *k *degree over the number of all nodes. Thus, the reliable GIN is a scale-free network as expected. Figure 4(b) also presents this GIN in a network view. For a clear view, Figure 4(c) in a network view shows another reliable GIN with *occ *= 12 and the percentage of *len *equal to 0.3. This GIN has 1,495 gene interactions. This GIN is shown here due to the suitable size for better view only. This GIN, as shown in Figure 4(c), indicates modularity property which is important to biological gene networks [23,24].

### Functional enrichment analysis for gene groups

Functional enrichment analysis is performed on a gene group. In this work, a gene group comprises those genes which are associated with a same transcription factor in a given GIN. In this way, we can further investigate the performance of the inferred GINs for transcription factor-gene regulations. Two GINs with the similar edge sizes are used here: an integrative reliable GIN with *occ *= 12 and the percentage of *len *equal to 0.25, and a PCC 1CC elu GIN with a threshold 0.8. The former has 27 gene groups, while the latter has 28 groups, if gene groups are required to have not less than 10 genes. Functional enrichment analysis of each gene group is performed using the enrichGO method in R [25] on Gene Ontology (GO) terms individually from biological processes, cellular components and molecular functions. In enrichGO, we use the Benjamini-Hochberg procedure to control the false discovery rate (q-value = 0.05) in multiple testing.

**Figure 5:**
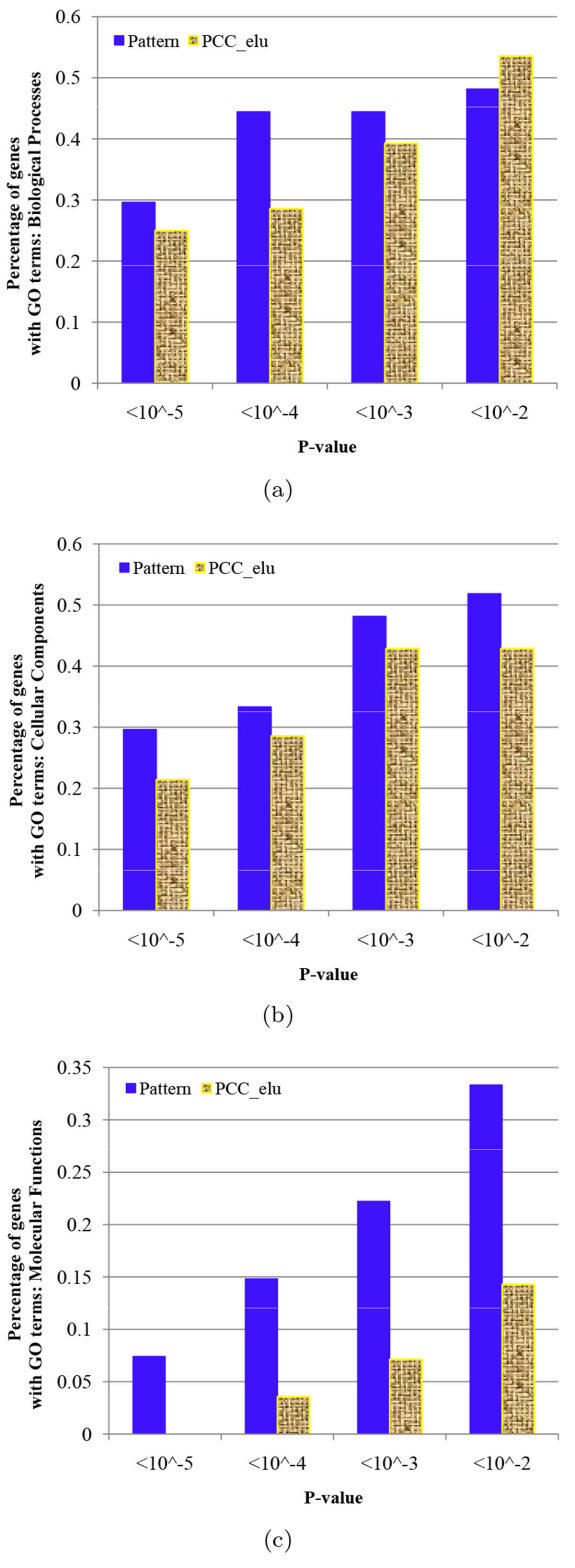
**Functional enrichment analysis: The percentage of gene groups with significant GO terms**. In each gene group, genes are associated with a same transcription factor in a given GIN. (a) GO terms: biological processes. (b) GO terms: cellular components. (c) GO terms: molecular functions. 'Pattern' represents an integrative reliable GIN. Y-axis represents the percentage of gene groups which have a GO term under corresponding p-value requirements over all gene groups whose size is not less than 10. Gene groups whose size is less than 10 are not used in functional enrichment analysis.

Then for each GIN, we calculate the percentage of gene groups which have significant GO terms under given p-values. We choose four different p-values, *i.e*., < 10^-2^, < 10^-3^, < 10^-4 ^and < 10^-5^. The result is shown in Figure 5, which suggests that the reliable GIN always has higher percentage than the PCC_1CC_elu GIN under smaller p-values. In particular, the percentage of the reliable GIN is much higher than that of the PCC_1CC_elu GIN in molecular functions (Figure 5(c)).

In functional enrichment analysis, several GO terms in a gene group may be significant with different subsets of genes under a given p-value. Thus in each gene group, we calculate the percentage of genes which are associated with a GO term under a p-value. The largest percentage in a gene group is used for this group. Given a p-value, we then calculate the mean and standard deviation of the largest gene percentage for all gene groups. The result is shown in Table 3. Again, the reliable GIN has much higher value than the PCC 1CC elu GIN. In a word, the reliable GIN is more functionally meaningful than the PCC 1CC elu GIN.

**Table 3:** Functional enrichment analysis: The statistics of the percentage of genes in each gene group where genes are associated with a same transcription factor in a given GIN.

p value	Biological Processes		*Cellular Components*		Molecular Functions	
	**reliable GIN**	**PCC elu^1 ^GIN**	***reliable GIN PCC***	***elu* ^1 ^ *GIN***	**reliable GIN**	**PCC elu^1 ^GIN**

< 0.00001	0.14 ± 0.23	0.09 ± 0.20	0.12 ± 0.20	0.10 ± 0.22	0.02 ± 0.08	0.00 ± 0.00

< 0.0001	0.23 ± 0.27	0.11 ± 0.21	0.14 ± 0.21	0.11 ± 0.22	0.04 ± 0.11	0.00 ± 0.02

< 0.001	0.27 ± 0.32	0.19 ± 0.29	0.22 ± 0.26	0.21 ± 0.31	0.08 ± 0.16	0.03 ± 0.12

< 0.01	0.33 ± 0.36	0.28 ± 0.32	0.31 ± 0.35	0.26 ± 0.37	0.14 ± 0.21	0.07 ± 0.19

^1^: the PCC 1CC elu GINs. In the format of XXX ± YYY, XXX(YYY) is the mean(standard deviation) of the percentages of genes in gene groups which have significant GO terms under a given p-value.

Each gene group may be associated with several significant GO terms. The largest percentage of genes is used for a gene group. If a gene group has no significant GO term, the percentage is 0.

### Case studies

#### Interesting patterns in biopathways

To show the usefulness of our method, we investigate several subsequential patterns of genes using the process below. We firstly obtained an integrative GIN with *occ *= 12 and *len *= 0.3. This parameter setting is used, on one hand, because it results in a reliable and small GIN for analysis; on the other hand, because a large GIN would produce subsequential patterns each with too many genes which cannot be easily investigated. Then, we filtered subsequential patterns of genes in each dataset by removing those genes which are not in the integrative GIN. After that, we select five subsequential patterns of genes for investigation. The five patterns, each from a dataset, are shown in Table 4.

**Table 4:** Five subsequential patterns for case studies.

ID	dataset	#genes1	genes	bio-pathway
1	alpha38	6/7	YGL111W, **YGR128C, YHR170W, YLR222C, YCR057C, YPL093W, YER006W**	Ribosome biogenesis in Eukaryotes

2	alpha30	6/10	**YGL116W**, YGR099W, **YGR109C, YIL026C, YLR103C**, YMR031C, YPL144W, YPR113W, **YDR113C, YER111C**	Cell cycle - yeast

3	cdc15	7/12	**YGL003C**, YBR009C, YIL140W, **YJL187C, YML027W, YMR199W**, YNL030W, YOL007C YOR114W, **YOR195W, YPL256C, YDL003W**	

4	cdc28	6/10	YGL027C, **YJL187C**, YLL022C, **YLR103C, YMR199W, YPL153C, YDL003W**, YDL018C YDR097C, **YFL008W**	

5	alpha	6/14	YBR089W, YKL032C, **YLR103C**, YML102W, **YCL061C**, YNL233W, YNL339C, **YPL256C **YPR202W, **YDL003W, YDL101C**, YBL003C YER001W, **YER111C**	

^1^: in this column, X/Y denotes that the subsequential pattern contains Y genes and × genes are in the corresponding bio-pathways. Genes in **bold **are contained in the bio-pathways.

**Figure 6:**
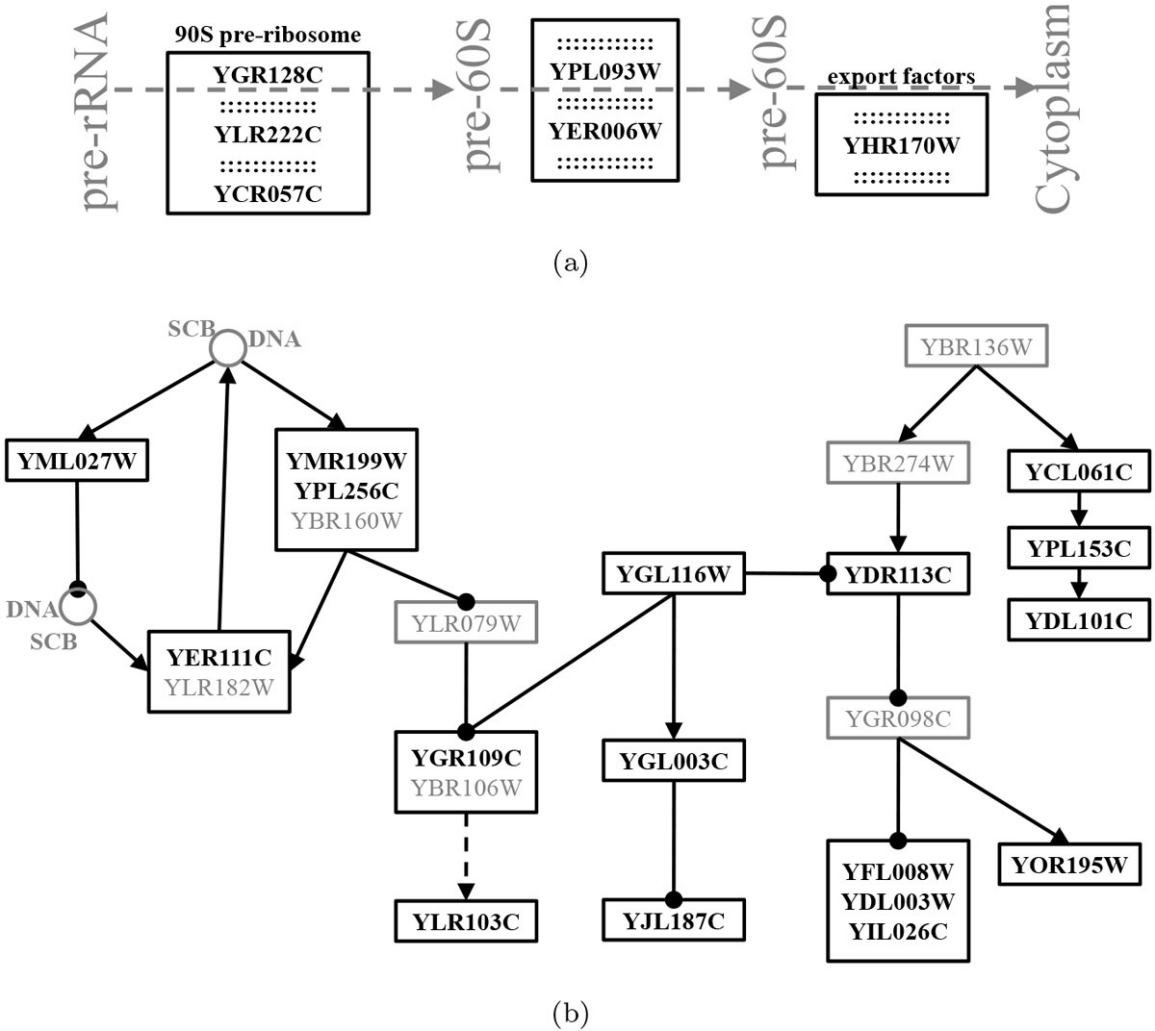
**The overlapping of five subsequential patterns of genes and the two bio-pathways**. (a) the overlapping of the first subsequential pattern in Table 4 and the bio-pathway of *Ribosome biogenesis in Eukaryotes*. (b) the overlapping of the last four subsequential patterns in Table 4 and the bio-pathway of *Cell cycle - yeast*. Genes in **bold **are those genes involved in the corresponding bio-pathways, while genes in gray are not covered by the four subsequential patterns. Arrows in **bold **denote molecular interactions or relations, while lines with big dots represent inhibitions.

The first pattern in Table 4 is from the *alpha38 *dataset. This pattern has 7 genes and 6 of them (in **bold **in Table 4) are in the bio-pathway of *Ribosome biogenesis in Eukaryotes*. How these genes are involved in the bio-pathway is shown in Figure 6(a). In Figure 6(a), the three genes--YGR128C, YLR222C and YCR057C--are involved in 90S pre-ribosome particle in *Ribosome biogenesis in Eukaryotes*, YPL093W and YER006W are critical to mature pre-60S, and YHR170W is an adapter protein to help to export pre-ribosomal units to cytoplasm. What is interesting is that *Ribosome biogenesis *is closely linked to such cellular activities as growth and division, while the datasets used in this work describe how eukaryotic yeast cells grow, mature and divide to produce daughter cells.

Since the datasets used in this work is about yeast cell cycle, we select another four subsequential patterns (as shown in Table 4 respectively from the *alpha30, cdc15, cdc28 *and *alpha*) for the bio-pathway of *Cell cycle - yeast*. Each of the four patterns has significant overlapping genes with the bio-pathway, and they totally have 17 genes in the bio-pathway. The relation of those 17 genes in the biopathway is shown in Figure 6(b). Figure 6 clearly illustrates that filtered patterns are useful to uncover those genes in a same bio-pathway.

#### The overlapping of a reliable GIN and 4 ribosomal protein complexes

**Figure 7:**
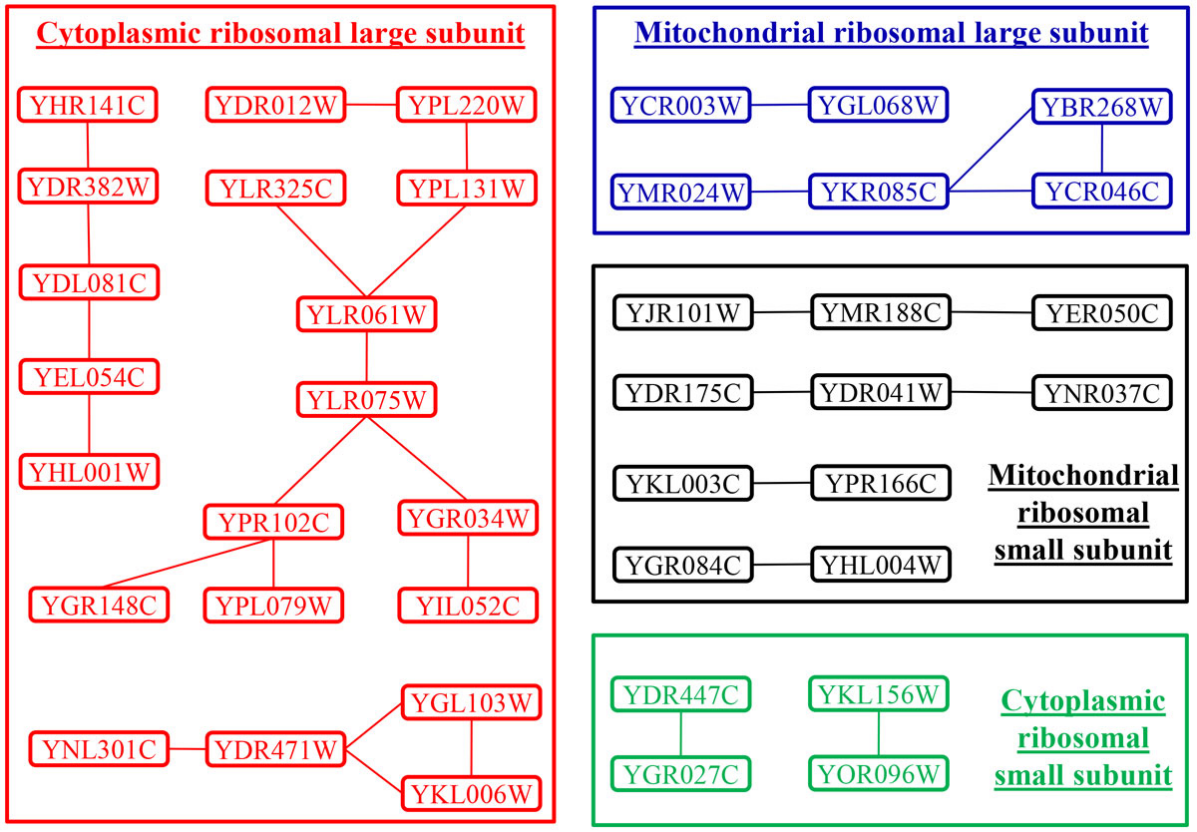
**The overlapping of a reliable GIN and 4 ribosomal protein complexes**. Better view in color. The proteins in red, green, blue and black are from the 4 ribosomal complexes respectively.

We also check the overlapping of 4 ribosomal protein complexes and a reliable GIN with *occ *= 12 and *len *= 0.25. The 4 complexes--*i.e., cytoplasmic ribosomal large subunit *with 81 proteins, *cytoplasmic ribosomal small subunit *with 57 proteins, *mitochondrial ribosomal large subunit *with 44 proteins, and *mitochondrial ribosomal small subunit *with 32 proteins--are downloaded from http://yeast-complexes.russelllab.org/complexview.pl?rm=download. They respectively have 38, 18, 24 and 17 proteins in the time-series datasets. For each complex, we get a subnetwork only with those edges whose proteins are both in the complex. These subnetworks are shown in Figure 7 with 20, 4, 6 and 10 proteins respectively for 4 complexes. It seems that the proteins in a same complex have significant connections in the reliable GIN. For example, the complex of *cytoplasmic ribosomal large subunit *have three connected subnetworks derived from the reliable GIN, demonstrating that many gene interactions in the reliable GIN are consistent with domain knowledge from protein complexes. Please note that our algorithm is not specifically designed for protein complex prediction and those genes in a pattern might indicate different properties of gene behaviors, such as in protein-protein interactions, biopathways, transcriptional regulations and/or other GO functions, while protein complexes are only one of these gene behaviors.

## Conclusion

In this work, we developed a framework of how to reconstruct a reliable gene interaction network from multiple time-course gene expression datasets. We proposed a novel subsequential pattern to capture potential gene interactions on each time-course gene expression dataset. We then reconstructed individual gene interaction networks using these patterns. After that, we built the reliable gene interaction network using those gene interactions which are conserved in individual gene interaction networks. We validated the reliable gene interaction network using three kinds of external data whose gene relation was determined by wet-lab experiments. The results demonstrated that our algorithm is substantially useful to decipher gene interaction networks from multiple time-course gene expression data, especially for less studied organisms where little knowledge is available except gene expression data.

## Competing interests

The authors declare that they have no competing interests.

## Authors' contributions

QL conceived the idea, designed the methods and performed the experiments. JL supervised the study. JL and RS participated in the analysis. QL drafted the manuscript. QL, RS and JL read and revised the manuscript. All authors approved the final version.

## Declarations

Publication charges for this article have been funded by an Australia Research Council Discovery Project (ARC DP130102124).

This article has been published as part of *BMC Genomics *Volume 16 Supplement 12, 2015: Joint 26th Genome Informatics Workshop and 14th International Conference on Bioinformatics: Genomics. The full contents of the supplement are available online at http://www.biomedcentral.com/bmcgenomics/supplements/16/S12.
